# Lower than expected hepatitis B virus infection prevalence among first generation Koreans in the U.S.: results of HBV screening in the Southern California Inland Empire

**DOI:** 10.1186/1471-2334-14-269

**Published:** 2014-05-17

**Authors:** Natali Navarro, Nelson Lim, Jiah Kim, Elliot Joo, Kendrick Che, Bruce Allen Runyon, Michel Henry Mendler

**Affiliations:** 1 Division of GI and Liver Disease, University of California at San Diego Health System, 200 W Arbor Drive #8413, San Diego, CA 92103-8413, USA; 2 Division of GI and Liver Diseases, Loma Linda University Medical Center, 11234 Anderson St., Loma Linda, CA 92354, USA

**Keywords:** Hepatitis B, Population screening, Korean immigrants, Hepatitis B carrier, Hepatitis B vaccination

## Abstract

**Background:**

Hepatitis B virus (HBV) infection is prevalent in Asian immigrants in the USA. California’s Inland Empire region has a population of approximately four million, including an estimated 19,000 first generation Koreans. Our aim was to screen these adult individuals to establish HBV serological diagnoses, educate, and establish linkage to care.

**Methods:**

A community-based program was conducted in Korean churches from 11/2009 to 2/2010. Subjects were asked to complete a HBV background related questionnaire, provided with HBV education, and tested for serum HBsAg, HBsAb and HBcAb. HBsAg positive subjects were tested for HBV quantitative DNA, HBeAg and HBeAb, counseled and directed to healthcare providers. Subjects unexposed to HBV were invited to attend a HBV vaccination clinic.

**Results:**

A total of 973 first generation Koreans were screened, aged 52.3y (18-93y), M/F: 384/589. Most (75%) had a higher than high school education and were from Seoul (62.2%). By questionnaire, 24.7% stated they had been vaccinated against HBV. The serological diagnoses were: HBV infected (3.0%), immune due to natural infection (35.7%), susceptible (20.1%), immune due to vaccination (40.3%), and other (0.9%). Men had a higher infection prevalence (4.9% vs. 1.7%, p = 0.004) and a lower vaccination rate (34.6% vs. 44.0%, p = 0.004) compared to women. Self-reports of immunization status were incorrect for 35.1% of subjects.

**Conclusions:**

This large screening study in first generation Koreans in Southern California demonstrates: 1) a lower than expected HBV prevalence (3%), 2) a continued need for vaccination, and 3) a need for screening despite a reported history of vaccination.

## Background

Hepatitis B is caused by the hepatitis B virus (HBV), which is transmitted through exposure to infectious body fluids. Most HBV carriers remain contagious for the rest of their lives. Chronic infection can lead to cirrhosis and liver cancer. An estimated 350 million people are diagnosed with hepatitis B worldwide [1]. Of those with chronic HBV infection 20% die from liver related disease complications.

In the U.S. the chronic HBV infection prevalence, defined by a positive hepatitis B surface antigen (HBsAg), is estimated to be 0.3-0.5% [2]. This low rate is partly attributed to HBV vaccination and prevention strategies established nationally since 1991, ahead of the World Health Organization’s (WHO) recommendations. The WHO’s resolution to include HBV immunization in childhood vaccination programs worldwide began in 1992 [3]. By WHO reports, childhood HBV vaccination policies were in place in 93% of its member states by 2012 [4]. Yet, HBV infection persists as a global health challenge with the prevalence remaining high, especially in Asia where most infections are acquired perinatally or during early childhood.

South Korea is currently classified by the Centers for Disease Control and Prevention (CDC) as an endemic area (≥8%) for HBV infection. Prior to the inclusion of the HBV vaccine in the Korean mandatory immunization program in 1995, the prevalence of HBV was estimated at 8.2% [5]. However, following WHO guidelines, the implementation of vaccination policies in South Korea have resulted in a decrease of HBV prevalence. In 1998 the National Health and Nutrition Survey reported HBV infection in South Korea to be 4.6% [6]. Data from the same survey in 2007 indicated a decline to 3.7% [7,8]. Similarly significant decreases in prevalence have been reported across Asia as a result of hepatitis B vaccination policies [9-11].

In the U.S. the CDC estimates two-thirds of those chronically infected with HBV are foreign-born and about half are of Asian descent [12]. Accordingly, current AASLD guidelines recommend HBV screening for Asians, yet recent survey data [13-15] shows among insured Asian-American patients only about half are tested. These studies also show healthcare providers do not perceive patients’ racial background or country of origin with high endemicity as risk factors for HBV, and that among primary care providers serving large American Asian communities only two thirds understand the serological tests required to identify infection. HBV prevalence studies in Asian American adults consistently identify lack of knowledge regarding serological status, the importance of being tested, disease transmission and prevention [16,17]. Community outreach programs have proven to be effective at increasing HBV awareness, identifying patients with HBV infections and offering services for linkage to care, especially for those lacking insurance or showing low cultural affinity to the US healthcare system [18].

The combination of high HBV prevalence and low screening reports is a significant health concern in California as one of the top five states with the largest populations of Asians, 63% of which are foreign-born [19]. The 2007–2011 American Community Survey (ACS) estimates almost 24,000 residents in the Inland Empire (Urban area in Southern California consisting of Riverside County and San Bernardino County) identify themselves as of Korean origin or descent, 19,000 identify as 1st generation (foreign-born) Korean Americans [20]. Loma Linda University Medical Center serves the Inland Empire and a significant number of the Asian American residents of the area.

While there is a reasonable expectation of greater HBV prevalence among immigrants compared to the general U.S. population, there is no data regarding HBV prevalence among Koreans in the Inland Empire. The common presumption that HBsAg seroprevalence of foreign-born U.S. residents is similar to HBV endemicity in their country of origin is a complex premise due to the variation in rates recently reported. The aim of this study was to screen at least 1,000 adult Koreans in the Inland Empire to establish HBV serological diagnoses of infection and immunity, educate, and establish linkage to care for vaccination and consideration of treatment.

## Methods

We established a community-screening program within local Korean communities. Subjects were screened for hepatitis B at Korean Churches from November 2009 through February 2010. Religious congregations were chosen because of their known function as spiritual and social centers, providing easy access to large numbers of the population of interest at single sites. Advertisements highlighting the importance of hepatitis B testing and details were placed in Korean newspapers and newsletters several weeks prior to each event to target non-church attendees and subjects from other congregations in the area. Flyers were also posted at churches and local primary health clinics predominantly serving Koreans.

A bilingual Korean nurse (JK) led the events. Screening was preceded by a brief lecture on hepatitis B. Educational materials on hepatitis B were distributed to all attendees. Subjects completed a brief self-administered questionnaire and provided contact information for result notification. The questionnaire included variables such as age, gender, providence of origin, occupation, alcohol use, and prior hepatitis B testing and vaccination history. The questions were limited in scope so as not to be intrusive, time consuming, or culturally insensitive. Participants were encouraged to fill the questionnaires but its completeness was not a pre-requisite to screening. The questionnaire and all other study documentation were made available in English and Korean for the participants to select based on language preference.

Blood samples were obtained by venipuncture for standard diagnostic serologies: HBsAg, antiHBs, and anti-HBc (AMA Laboratory, Monrovia, CA, USA). Test results were used to place patients in serological profiles: 1) Infected (HBsAg positive, antiHBs negative, anti-HBc positive), Immune due to natural infection (HBsAg negative, antiHBs positive, anti-HBc positive), Susceptible (HBsAg negative, antiHBs negative, anti-HBc negative), Immune due to vaccination (HBsAg negative, antiHBs positive, anti-HBc negative), and Other/further testing required (HBsAg negative, antiHBs negative, anti-HBc positive). The processing laboratory was instructed to retain blood for all individuals to subsequently reflex HBsAg positive samples to Anti-HBe, HBeAg and HBV DNA quantitative PCR (Roche COBAS® TaqMan. LOQ 29 IU/ml) tests.

A letter describing test results and their interpretation (MM), along medical recommendations (MM) was mailed to each participant. Patients with HBsAg positive results also received result phone notification and initial counseling by the study nurse (JK). A special low-cost clinic was established for subjects without access to healthcare with a discounted total fee of $326.46 for hepatology consultation, additional laboratory tests (CBC, PT/INR/CMP, AFP), and liver ultrasound. Similarly, a special low-cost HBV vaccination clinic was created for those subjects unexposed to HBV, $108 for vaccine and booster. Results were reported to the population at large via an advertisement and report in the area’s largest circulation Korean newspaper. All cases were reported to the US Department of Health & Human Services. All participants provided written informed consent and the study was performed according to the World Medical Association Declaration of Helsinki. All study activities were approved by the Institutional Review Board at Loma Linda University (OSR#59204).

Analysis of the data was performed with statistics package SPSS v21.0 (SPSS Inc., Chicago, Ill., USA). Parametric data is expressed as mean (±SD), non-parametric is noted as median and range. Frequency is given as (N). Tests of significance report p-values. One Sample *t*-test was used to calculate prevalence and confidence intervals (95% CI). Two Sample *t*-test was used to examine differences in age between male and female subjects. Chi-square (*X*^2^) test was used to test if significant variables were different across groups.

## Results

### Subject accrual and demographics

We conducted 10 screening events across 9 Korean churches of varying denominations in the Inland Empire during the screening period (3.6 months). A total of 1,007 subjects were screened for HBV. Most (973) reported being born in Korea and had residence concentrated in 3 counties: San Bernardino (51.4%), LA (23.2%), Riverside (21.8%). We report on this first generation Korean American group.

**Figure 1:**
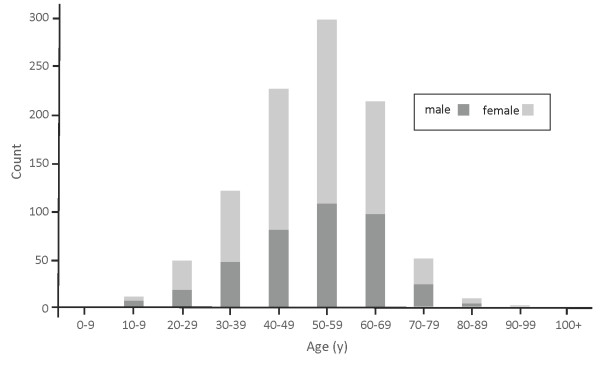
Overall study sample age distribution and gender stratification among 973 first generation Korean adults screened for Hepatitis B in the Inland Empire during 2009–2010.

The median age among subjects was 52.3y (18-93y), and 60.5% (589) were females. Age distributions according to gender (Figure 1, Table 1) were not significantly different (p = 0.128). More than half (62.2%) of participants were from the Seoul metropolitan area and 78.0% reported having immigrated to the U.S. before the year 2000. Regardless of long-term residence in this country, 97.1% of participants preferred Korean as the language for communication during screening and for result notification. Most participants reported greater than high school education (75%), while only 6% reported less than high school education (Table 2). A large number of participants declined to disclose their occupation (356), and of those who did, 47.2% (291) declared unemployment or limited income (student, homemaker, retired).

**Table 1:** Observed frequencies of HBsAg positive diagnosis stratified by age and gender among 973 first generation Korean adults screened for hepatitis B in the Inland Empire during 2009-2010

	**Male**			**Female**		
**Age (y)**	**HBsAg (+)**	**Sample population**	**Age specific prevalence (%)**	**HBsAg (+)**	**Sample population**	**Age specific prevalence (%)**
**0-9**	0	0	0.0	0	0	0.0
**10-19**	0	7	0.0	0	4	0.0
**20-29**	2	18	11.0	0	30	0.0
**30-39**	2	47	4.0	2	73	3.0
**40-49**	3	80	4.0	5	145	3.0
**50-59**	8	107	7.0	2	189	1.0
**60-69**	4	96	4.0	1	116	1.0
**70-79**	0	24	0.0	0	26	0.0
**80-89**	0	4	0.0	0	5	0.0
**90-99**	0	1	0.0	0	1	0.0
**100+**	0	0	0.0	0	0	0.0
**Total**	19	384	4.9	10	589	1.7

**Table 2:** Observed frequencies for serological diagnoses stratified by education level among 973 first generation Korean adults screened for hepatitis B in the Inland Empire during 2009–2010

**Education level**	**Infected**	**Immune due to natural infection**	**Susceptible**	**Immune due to vaccination**	**Other/further testing**	**Total**
**Declined to answer**	0	13 (3.7%)	5 (2.6%)	17 (4.3%)	0	35 (3.6%)
**Less than High School**	1 (3.5%)	35 (10.1%)	10 (5.1%)	10 (2.6%)	2 (22.2%)	58 (6.0%)
**High school**	5 (17.2%)	59 (17.0%)	31 (15.8%)	51 (13.0%)	4 (44.4%)	150 (15.4%)
**More than High School**	23 (79.3%)	240 (69.2%)	150 (76.5%)	314 (80.1%)	3 (33.3%)	730 (75.0%)
**Total**	29 (100%)	347 (100%)	196 (100%)	392 (100%)	9 (100%)	973 (100%)

Almost all subjects (95.5%) denied either recent alcohol consumption (701) or declared only occasional use (229). Most participants denied knowledge of personal (82.3%) or family (76.8%) history of liver disease, and denied being previously tested for viral hepatitis (61.5%). Across the entire study sample, 24.7% (240) declared they had been vaccinated against HBV.

### Serological diagnoses

The prevalence of hepatitis B infection was 3.0% (1.9-4.0%). Immunity due to natural infection was seen in 35.7% (32.6-38.7%) of participants. Susceptible individuals constituted 20.1% (17.6-22.7%) of participants, while 40.3% (37.2-43.4%) had immunity due to vaccination. A small number of 0.9% (0.3-1.5%) of subjects were exposed but required further testing due to multiple interpretations of the results. Among the 29 infected subjects the median DNA quantitative PCR was 285 IU/ml (29 to ≥ 110,000,000 IU/ml): < 2,000 IU/ml in 17, 2000–20,000 IU/ml in 3, and >20,000 IU/ml in 8.

### Serological and demographic correlations

HBV prevalence was higher in males compared to females (4.9% vs. 1.7%, p = 0.004). The median age among those infected was 50 y (26-63y) with 23 of the 29 cases concentrated in the 40–69 age range and only 2 infected participants under 30 years old (Table 1). The majority (27) of those infected denied consuming alcohol (17) or declared only occasional use (10). The HBsAg positive group also denied knowledge of personal (72.4%) or family (58.6%) history of liver disease. Despite these reports, there was a higher proportion of HBsAg positive participants among subjects with a family history of liver disease compared to those without (6.3% vs. 2.3%, p = 0.023).

Almost half (13) of those infected stated this was the first time they had been tested for hepatitis B, and 5 reported having received vaccination. Most of the infected participants (20) were HBeAg negative with median DNA quantitative PCR of 164 IU/ml (29–18,019 IU/ml), while 8 cases were HBeAg positive with median DNA quantitative PCR of ≥ 110,000,000 IU/ml (36,040 to ≥ 110,000,000 IU/ml). Further testing was not performed for one of the HBsAg positive subjects due to low sample volume.

Of the 29 infected participants only 1 had health insurance. Despite this barrier, 2 of the 10 infected women and 6 of the 19 men had effective follow-up visits, for a total of 27.5% (8) success in linkage to care. On average, 78 days passed between result notification and appointment in our hepatology clinic for evaluation with further testing and ultrasound. Of the 8 participants seen in clinic half did not require treatment. The other half was provided guidance on patient medication assistance programs and started antiretroviral therapy.

The vaccination rate was higher in women as compared to men (44.0% vs. 34.6%, p. =0.004). The vaccination rate was significantly different across self-reported education levels (p < 0.001) (Table 2). The vaccination rate was 43.0% in those declaring “More than high school” education, 34.0% in those that graduated high school, and only 17.2% in those who had not.

There was a statistically significant (p < 0.001) discrepancy between immunity due to vaccination based on serological results and self-declared vaccination history across all participants (Table 3). Of the 240 subjects thought to have been previously vaccinated, there was a significant (p < 0.001) disparity with serological markers: 60.4% (145) had serological evidence of immunity by vaccine, 26.3% (63) had immunity due to natural infection, 10.8% (26) were susceptible, and 2.1% (5) were infected. 41.8% (199) of subjects reporting not being vaccinated (476) had in fact serological evidence of immunity by vaccine. The overall rate of incorrect report of immunization status was 35.1% (342), differing across education levels (p = 0.008) but not between genders (p = 0.888). Most (74.3%) of susceptible individuals requiring vaccination or follow up for additional tests did not have medical insurance. Only 1 participant followed the recommendation for vaccination and 1 for further testing.

**Table 3:** Observed frequencies for serological diagnoses stratified by declared HBV vaccination status among 973 first generation Korean adults screened for hepatitis B in the Inland Empire during 2009–2010

**Declared vaccination**	**Infected**	**Immune due to natural infection**	**Susceptible**	**Immune due to vaccination**	**Other/further testing**	**Total**
**Yes**	5 (17.2%)	63 (18.2%)	26 (13.3%)	145 (37.0%)	1 (11.1%)	240 (24.7%)
**No**	16 (55.2%)	199 (57.3%)	127 (64.8%)	127 (32.4%)	7 (77.8%)	476 (48.9%)
**Do not know**	8 (27.6%)	85 (24.5%)	43 (21.9%)	120 (30.6%)	1 (11.1%)	257 (26.4%)
**Total**	29 (100%)	347 (100%)	196 (100%)	392 (100%)	9 (100%)	973 (100%)

## Discussion

This study examined the prevalence hepatitis B serological diagnoses in first generation adult Koreans attending houses of worship in the Southern California Inland Empire. When compared with the most recent ACS data [21] on East Asian residents in San Bernardino and Riverside, our study sample was similar in mean age, proportion immigrating before 2000, education level, male to female ratio, and preference for using their native language. Consequently the prevalence found in this study is believed to be an accurate estimate to that of the first generation Koreans residing in the Inland Empire.

The 3.0% HBV prevalence is lower than the ≥8% endemic rate described by the CDC but closer to current reports of 3.7% out of Korea [8]. A recent study [22] examining a similar but mixed Korean and Chinese population in Southern California also found a lower than expected HBV prevalence of 3.9% among Koreans and discrepancies between reported vaccination and immunity by serological evidence. Our data is also comparable to this study in terms of demographics such as age, gender ratio, and proportion of foreign-born individuals. Our cohort is however perhaps more homogeneous, recruited solely by a “grass-roots” approach within attendees at religious congregations, without direct recruitment of subjects in or to medical facilities. Recent data from the California Health Interview Survey show that Korean Americans have the highest uninsured rate among Asian Americans in California [23]. With as many as 70% of Koreans in the U.S. attending religious services regularly, most being first generation immigrants [24], we believe that religious congregations are therefore optimal locations for health screenings of this type.

The HBV prevalence was higher for men than for women, congruent with previous studies showing gender difference in seroconversion and HBsAb production after infection [22,25-27]. Both the prevalence of HBsAg and age distribution of the study sample were concentrated in the 40–59 year age group. As expected, subjects <20 years old had the lowest HBV prevalence. Subjects born after the introduction of the universal vaccination reflect the impact of these programs. However this age group (11) was not well represented in our sample. South Korea’s population proportion of this age bracket is much higher than in our sample (33.5% vs. 1.1%).

Analysis of our data leads us to believe that Korean province of origin and reported education level play a greater role in the observed prevalence in this study. Two thirds of all participants stated migrating from the capital city of Seoul, the country’s biggest urban and industrial center [28]. Areas of urban development such as Seoul see not only the highest proportions of tertiary education but are often the epicenter of national policies such as vaccination programs [29]. The education level for most study participants of studies beyond high school (75%) was comparable to that of Seoul (69%) and higher than the reported average secondary level of education for adults in all South Korea [30]. As previously reported, both education level and access to care in metropolitan households are strongly associated with decreased prevalence, increase vaccination rates and awareness of hepatitis B [31,32]. The authors recognize some questions were not perceived as suitable for the public setting due to social stigma, as reflected in the reluctance to answer items in our questionnaire pertaining to sensitive information. Consequently, it is difficult to associate our findings to other factors.

Nonetheless, the lower HBV prevalence points to a closer examination of CDC and WHO travel guidelines [5]. New epidemiological data can serve to reclassify South Korea to intermediate hepatitis B prevalence level, reflecting successful universal HBV vaccination programs that have had a documented impact on the hepatitis B infection decline in China, Taiwan and South Korea [9-11]. The reported 0.2% HBV prevalence for those < 18 years [33] in South Korea is comparable to that of the U.S. general population. On the other hand HBV prevalence remains as high as 6.2% for adults aged 50–60 years old in Asia [7], suggesting that age-based cohort screening for immigrants is recommended.

Subjects in this study had significant discrepancies between vaccination based on serological results and self-declared vaccination history. These results highlight the importance of complete serological screening among immigrants before recommending immunization, as well as serological follow up to confirm successful immunization. A recently issued IOM action plan for Hepatitis B prevention and management [34] is consistent with cost-effectiveness studies favoring the ‘Screen and Treat’ model [35-37] and the incorrect report of immunization status findings in this cross sectional prevalence study. Furthermore, our results illustrate providers and policy makers should consider the new prevalence estimates when providing services for foreign-born patients.

Hepatitis B screening should be approached considering the prevalence linked to patient’s country of origin. However, there is great need for revision of historically reported rates across the world in order to assess a more accurate infection risk among immigrants. Barriers such as poor HBV knowledge, low socioeconomic and educational background are associated with low rates of self-initiated screening across immigrants from nations with >2.0% HBV prevalence [28]. These factors should lead to targeted screening. Considering the growing number of Asian immigrants, California faces an especially complex challenge among its foreign-born residents due to language barrier, lack of access or understanding the healthcare system, cultural beliefs, denial or unawareness of increased risk, social stigma, concern about cost and results.

## Conclusion

The hepatitis B infection prevalence among first generation Korean immigrants in the Inland Empire may be lower than expected based on previous reports from the CDC and WHO. Self-report of hepatitis B immunization status is often inaccurate and can lead to additional costs, unnecessary vaccination, and dangerous perception of prevention among contagious carriers. Despite the lower than expected HBV prevalence, an infection rate >2% indicates that this population is still at risk and should be screened. Complete serological testing can lead to optimal management of those individuals at risk for hepatitis B.

## Competing interests

The authors declare that they have no competing interests.

## Authors’ contributions

NN: Carried out data quality evaluation, performed the statistical analysis and drafted the manuscript. NL, JK, EJ, KC, BAR: Participated in the design of the study and revising it critically for important intellectual content. MHM: Conceived of the study, participated in its design and coordination and helped to draft the manuscript. All authors read and approved the final manuscript.

## Pre-publication history

The pre-publication history for this paper can be accessed here:

http://www.biomedcentral.com/1471-2334/14/269/prepub
